# The specific targeting of immune regulation: T-cell responses against Indoleamine 2,3-dioxygenase

**DOI:** 10.1007/s00262-012-1234-4

**Published:** 2012-03-03

**Authors:** Mads Hald Andersen

**Affiliations:** Department of Hematology, Center for Cancer Immune Therapy (CCIT), Copenhagen University Hospital, Herlev, Herlev Ringvej 75, 2730 Herlev, Denmark

**Keywords:** IDO, Antigen, Immune suppression, Supporter T cells, PIVAC 11

## Abstract

Indoleamine 2,3-dioxygenase (IDO) is an immunoregulatory enzyme that is implicated in suppressing T-cell immunity in many settings including cancer. In recent years, we have described spontaneous CD8^+^ as well as CD4^+^ T-cell reactivity against IDO in the tumor microenvironment of different cancer patients as well as in the peripheral blood of both cancer patients and to a lesser extent in healthy donors. We have demonstrated that IDO-reactive CD8^+^ T cells were peptide-specific, cytotoxic effector cells, which are able to recognize and kill IDO-expressing cells including tumor cells as well as dendritic cells. Consequently, IDO may serve as a widely applicable target for immunotherapeutic strategies with a completely different function as well as expression pattern compared to previously described antigens. IDO constitutes a significant counter-regulatory mechanism induced by pro-inflammatory signals, and IDO-based immunotherapy may consequently be synergistic with additional immunotherapy. In this regard, we have shown that the presence of IDO-specific T cells boosted immunity against CMV and tumor antigens by eliminating IDO^+^ suppressive cells and changing the regulatory microenvironment. The current review summarizes current knowledge of IDO as a T-cell antigen, reports the initial results that are suggesting a general function of IDO-specific T cells in immunoregulation, and discusses future opportunities.

## IDO and immune suppression

The immune system is delicately balanced between immunity and tolerance to protect the host from pathogens while minimizing local damage to tissues. Indoleamine 2,3-dioxygenase (IDO) is an endogenous molecular mechanism that contributes to this immune regulation in a variety of settings. IDO seems to be critical in limiting potentially exaggerated inflammatory reactions in response to danger signals [33] and in assisting regulatory T-cell effector function [32]. In addition, IDO is an important component of a regulatory system that allows long-term control of immune homeostasis as may be required by tolerance to self or during pregnancy [27].

IDO is a major inhibitor of the effector phase of the immune response [45, 50]. IDO expression can suppress effector T cells directly by degradation of the essential amino acid tryptophan. Some of the biological effect of IDO is mediated through local depletion of tryptophan, but is in addition mediated via immune modulatory tryptophan metabolites [4, 30]. Thus, regulation of tryptophan metabolism by IDO in dendritic cells (DC) is a highly adaptable modulator of immunity. When IDO^+^ DC are injected in vivo, they create suppression and anergy in antigen-specific T cells in the LN draining the injection site [3, 25]. Effector T cells starved of tryptophan are unable to proliferate and go into G1 cell cycle arrest [25]. An IDO-responsive signaling system in T cells has been identified, comprising the stress kinase GC non-derepressing 2 kinase (GCN2). GCN2 responds to elevations in uncharged tRNA, as would occur if the T cell were deprived of tryptophan [24].

Another effect of IDO is mediated through enhancement of local Treg-mediated immune suppression. Constitutive IDO expression in DC provides T cells with regulatory properties that block T-cell responses to antigenic stimulation [24]. The B7 receptors on IDO^+^ DC bind to CTLA4 on Tregs causing them to proliferate and induce antigen-specific anergy. Thus, IDO does not only suppress effector T cells directly but also influence Tregs bystander suppressor activity [2, 32, 39].

It has been described that exposure of Tregs to pro-inflammatory cytokines like IL-6 induce reprogramming of mature Tregs to acquire a phenotype resembling pro-inflammatory Th17 cells [6, 49, 51]. IDO plays a vital role in this conversion [2, 39]. IDO stimulates Treg bystander suppressor activity and simultaneously blocks the IL-6 production that is required to convert Tregs into Th17-like T cells [2, 39]. The phenotype of reprogrammed Tregs after IDO-blocking have been described as similar to that of “polyfunctional” T-helper cells co-expressing IL-17, IL-22, IL-2 as well as TNF-α [39]. Thus, IDO suppression of pro-inflammatory processes may dominantly block effector T-cell responses to antigens encountered. Conversely, absence of IDO activity may not elicit local Treg suppression even when strong pro-inflammatory stimuli are present.

Finally, it was recently shown that IDO has a non-enzymic function that contributes to TGF-β driven tolerance in non-inflammatory contexts [29].

## IDO and cancer

IDO expression is widely deregulated in cancer patients. IDO may contribute in a critical manner to inhibit or terminate inflammation and are highly overexpressed in cancer [14, 22].

In cancer patients, IDO elevation occurs in a subset of plasmacytoid DC in tumor-draining lymph nodes [26]. In addition, IDO may be expressed within the tumor by tumor cells as well as tumor stromal cells, where it inhibits the effector phase of immune responses [45]. Activation of IDO in either tumor cells or nodal regulatory DC each appears to be sufficient to facilitate immune escape of tumors [24]. In this regard, it has been described that expression of IDO in tumor cells is associated with an impaired prognosis [46]. In a murine model, it was observed that tumor cells transfected with IDO became resistant to immune eradication, even in mice in which a fully protective immune response had been established by immunization [45]. IDO-expressing CD19^+^ plasmacytoid DC isolated from tumor-draining LN mediate profound immune suppression and T-cell anergy in vivo [25, 37], whereas plasmacytoid DC from normal LNs and spleen do not express IDO. In this respect, it should be noted that very few cells constitutively express IDO in normal lymphoid tissue except in the gut. It is believed that constitutive IDO expression in DC in tumor-draining LN is induced by stimulation from Tregs migrating from the tumor to the draining LN. Tregs have been shown to induce IDO via cell-surface expression of CTLA-4 [44]. The induction of IDO converts the tumor-draining LN from an immunizing into a tolerizing milieu.

All in all, IDO is a critical cellular factor contributing to immune suppression and as such is a crucial mechanism in cancer. Hence, IDO has become a very attractive target for the design of new anticancer drugs and several IDO inhibitors are under investigation in preclinical as well as in clinical studies [16]. In particular, the compound 1-methyl-tryptophan (1MT) has been widely studied as an inhibitor of IDO activity. Interestingly, recent studies have shown that the racemer D-1-MT has superior antitumor activity compared to the racemer L-1-MT [13]. A novel indoleamine 2,3-dioxygenase (IDO)-like protein designated IDO2 was recently discovered [20]. IDO2 functions like IDO in tryptophan catabolism, but it has been found that D-1MT but not the L-1MT isomer selectively and potently inhibits IDO2 activity suggesting that IDO2 activity may have a role in the inhibition of immune responses to tumors. In this respect, IDO2 expression has been found in human tumors, including gastric, colon, renal, and in pancreatic tumors IDO2 expression have been found both in tumor cells as well as in immune cells in tumor-draining LN [47]. It is not yet known to what extent each isoform of IDO contributes to tumor-related immune suppression and how much clinical benefit (or autoimmune toxicity) targeting one isoform over another confers. Another unknown is whether IDO inhibitors influence other pathways not directly linked to IDO.

## CD8 responses against IDO

**Fig. 1:**
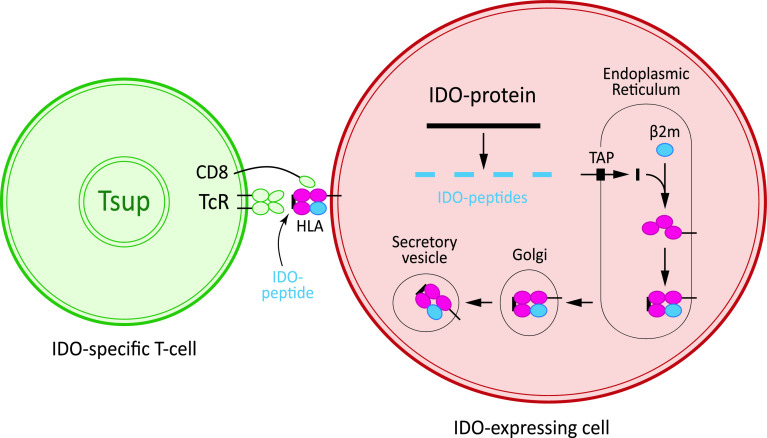
Principle of the processing pathway of IDO peptides by IDO-expressing cells (*red*), for example, tumor cells or dendritic cells and the subsequent recognition by specific CD8 T cells (*green*; here entitled a “supporter T cell” (Tsup).The epitopes recognized by the T cells are short IDO-derived peptides resulting from the degradation of intracellular IDO protein, which are presented on the cell surface of HLA molecules. T cells receive an activation signal through their T-cell receptor complex, leading to a variety of functional consequences, including release of cytokines and cytotoxic molecules

Despite the fact that neoplastic transformation is associated with the expression of immunogenic antigens, the immune system often fails to respond effectively and becomes tolerant toward these antigens [21]. As described above IDO plays a critical role in the tolerance induction and immune suppression of anti-cancer immune responses. We sat out to determine if and how IDO itself serve as target for specific T-cell responses, which may be exploited for immune therapy. This was done by identifying and characterizing specific T cells spontaneously present among peripheral blood mononuclear cells (PBMC) isolated from cancer patients of different origin. In this regard, we described that peptides comprised in the IDO protein sequence are spontaneously recognized by cytotoxic T cells (CTL) in cancer patients (Fig. 1) [40].

First, we identified HLA-restricted peptides within the IDO protein to which spontaneous T-cell reactivity were detected in patients suffering from unrelated tumor types, i.e., melanoma, renal cell carcinoma and breast cancer by flow cytometry using HLA/peptide tetramers as well as in ELISPOT assays after in vitro stimulation but also in direct ex vivo assays. Such IDO-reactive CD8^+^ T cells were peptide-specific, cytotoxic effector cells. Thus, IDO-specific T cells effectively lysed IDO^+^ cancer cell lines of different origin, such us colon carcinoma, melanoma, and breast cancer as well as directly ex vivo enriched leukemia cells. IDO driven immune suppression is a general mechanism that has been described in a variety of human cancers and the immune responses against IDO seem likewise to be relevant in cancers of unrelated origin, which emphasize the immunotherapeutic potential of IDO. However, even more distinctive was our finding that IDO-specific CTL recognized and killed IDO^+^, mature DC; hence, IDO-specific T cells were in addition able to kill immune-regulatory cells*.* We could at first not detect spontaneous responses against IDO in the control group of healthy individuals. Thus, although IDO has immune suppressive functions, the constitutive up regulation of IDO expression in cancer patients seemed to induce IDO-specific T-cell responses.

**Fig. 2:**
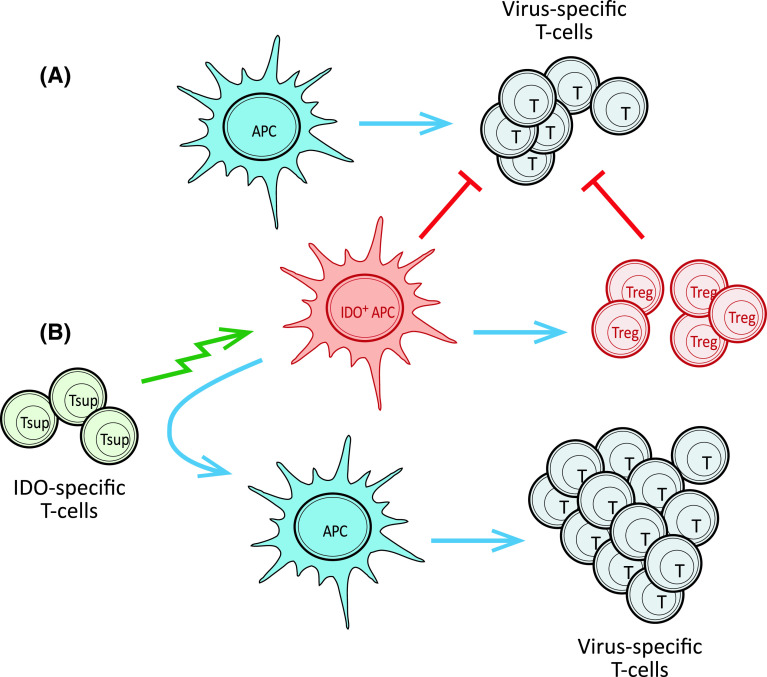
IDO-specific T cells are able to boost specific immunity against virus or tumor antigens in human PBMC. **a** When stimulating PBMC with a known HLA-restricted T-cell virus epitope and IL-2, epitope-specific T cells begin to expand due to activation by antigen presenting cells (APC). In response to the subsequent production of cytokines like INF-γ, IDO expression is induced and IDO-expressing APC inhibit further expansion of virus-specific T cells both directly and indirectly through activation of Tregs. **b** The addition of cytotoxic, IDO-specific T cells (Tsup) removes immune suppressive cells from the PBMC culture thereby facilitating further expansion of virus-specific T cells

IDO is playing a crucial role in immune regulation and is inducible under normal physiological conditions. Thus, we found the apparent lack of tolerance against IDO intriguing, since it suggested a more general role of IDO-specific T cells in the regulation of the immune system. We hypothesized that such cells could take part in the control of immune homeostasis; IDO-specific CD8^+^ T cells could play an important role by eliminating IDO^+^ cells thereby suppressing and/or delaying local immune suppression. Hence, we continued our search for possible IDO-specific T-cell responses in healthy donors and found that circulating IDO-specific, cytotoxic CD8^+^ T cells indeed were present in healthy donors although not as frequent as in patients with cancer [41]. Furthermore, we were able to directly link the up regulation of IDO with IDO-specific T cells by showing that the addition of IDO-inducing mediators like IFN-γ and CpG ODN generated measurable numbers of CD8^+^ IDO-specific T cells among PBMC. To examine a possible immune-regulatory effect of IDO-specific T cells, we examined their effect on T-cell immunity against viral or tumor-associated antigens. In this respect, we found that the presence of IDO-specific CD8^+^ T cells boosted CD8^+^ T-cell responses against other antigens probably by eliminating IDO^+^ suppressive cells (Fig. 2). Consequently, we suggested terming IDO-specific T cells “supporter T cells” (Tsup) due to their immune enhancing function [41].

IDO expression contributes to the strength and duration of a given immune response due to its inflammation-induced counter-regulatory function. Thus, any “supportive” effect of IDO-specific T cells on other immune cells may well be mediated in several direct and indirect manners. In this respect, the level of tryptophan was elevated, the frequency of Tregs decreased, and the frequency of IL-17 producing cells increased when IDO-specific T cells were present, which taken together suggest an overall decrease in IDO activity. Furthermore, IDO-specific T cells increased the overall production of both IL-6 as well as the other pro-inflammatory cytokine TNF-α. In contrast, we observed a decrease in IL-10. Another possible effect of IDO-specific T cells could be mediated through the metabolites of tryptophan, which have been shown to be directly toxic to CD8^+^ T cells and CD4^+^ Th1 cells [11], but not Th2 cells. Hence, increased IDO activity seems to tilt helper T-cell polarization toward a Th2 phenotype [48]. The presence of activated IDO-specific, cytotoxic T cells may screw the Th-response in a Th1-direction. Finally, it should be noted that IDO^+^ cells may be immune suppressive by other means than by the expression of IDO. Hence, the same cells might express, for example, Arginase, PD-L1 or immune-regulatory cytokines (e.g., IL-10 and TGF-β). Hence, IDO-specific, cytotoxic T cells may not only reduce IDO-mediated suppression directly but in addition further immune suppression mediated by IDO^+^ regulatory cells.

Recently, we identified spontaneous CD8^+^ T-cell reactivity against the IDO analogue IDO2 in peripheral blood of both healthy donors and cancer patients [42]. Furthermore, we confirmed that IDO2-reactive CD8^+^ T cells were peptide-specific, cytotoxic effector T cells. Hence, isolated and expanded IDO2-specific T cells effectively lysed cancer cell lines of different origin, that is, colon carcinoma cells as well as breast cancer cells. However, IDO2-specific T cells did not seem to kill melanoma cells although they expressed IDO2. At least, we did not observe killing of three different IDO2^+^ melanoma cell lines. Likewise, IDO2-specific T cells did not seem to “support” other immune responses in the same way as IDO-specific, cytotoxic T cells. Hence, the function and potential role of the IDO2-specific class-I-restricted lymphocytes present in peripheral blood still need to be resolved.

## CD4 responses against IDO

We speculated that CD4^+^ IDO-specific T cells releasing pro-inflammatory cytokines may play a role in the early phases of an immune response as a counter-response to the induced immune suppression facilitated by IDO^+^ cells. Hence, IDO-specific Th1-cells may delay local immune suppression if the activation of an IDO-specific CD4^+^ Th1-response could overcome the immune suppressive actions of the IDO protein, which are otherwise a result of the early expression of IDO in maturing DC or macrophages. Hence, we went on to analyze if CD4^+^ T cells naturally recognized IDO. Indeed, identified detectable numbers of specific CD4^+^ T cells both in cancer patients as well as healthy individuals [23].

We found that such IDO-specific CD4^+^ T cells released INF-γ as well as TNF-α. Although, we were able to detect both INF-γ and TNF-α response toward IDO in healthy donors, the responses were more frequent in cancer patients. The cancer relevance of these CD4^+^ T cells were further underlined, since IDO-reacting T cells in addition react toward DC pulsed with IDO^+^ tumor lysates. Interestingly, we detected a correlation between patients harboring CD4 and CD8 responses against IDO, which that class-I- and class-II-restricted IDO responses co-develop.

Furthermore, we detected frequent IDO-specific CD4^+^ T-cell responses when examining for IL-17 release upon stimulation with the IDO-derived CD4 epitope. IL-17 has been the focus of great interest recently since the production of IL-17 is characterized to a subset of CD4^+^ T-helper cells (Th17 cells). One of the main roles of Th17 cells is believed to be promoting host defense against infectious agents. Th17 cells are thought to be particularly important in maintaining barrier immunity at mucosal surfaces such as in the lungs, gut, and skin [28]. Interestingly, IDO is expressed at high levels in the gastrointestinal tract, although its precise role in intestinal immunity is not well understood [7]. One could speculate that a fraction of the Th17 that are highly prevalent at the mucosal tissues of healthy individuals [28] is recognizing IDO; however, this is yet to be established. Additionally, it is well described that Th17 cells contribute to autoimmunity [6]. In cancer, Th17 cells might have a protective role in tumor immunopathology by promoting antitumor immunity. Tumor-infiltrating Th17 cells express other cytokines in addition to IL-17, which might be functionally relevant [18]. A large fraction of Th17 cells produce high levels of effector cytokines such as IL-2, INF-γ as well as TNF [51]. IDO-specific Th17 cells seemed to exhibit a similar effector T-cell cytokine profile [23]. We could in contrast not detect any release of the Th2 cytokine IL-4 in response to the IDO-derived peptide [23].

It was recently suggested that the Foxp3^+^ Treg cell lineage in addition to immune suppression have an unappreciated helper role [38]. These “Th17-like effector cells” were distinguished by their unique ability to deliver help immediately and spontaneously, without needing prior priming or pre-activation. It was suggested that these CD4 lineage cells correspond to a pool of constitutively primed “first responder” cells [38]. IDO plays an important role in this conversion of Foxp3^+^ Tregs to Th17-like effector cells [2, 39]. Thus, it is possible that IDO-specific T cells could in addition belong to a Foxp3^+^ lineage of constitutively primed “first responder” Th17-like T cells; however, it should be strengthen that this is speculation.

Naturally, some CD4-positive IDO-specific T cells could in addition be immune suppressive Tregs. It would be obvious that IDO-specific Tregs may enhance the IDO-mediated immune suppression protecting cells from an immune attack. In this regard, we have previously described specific regulatory CD8^+^ T cells in cancer patients, which recognized the immune suppressive Heme Oxygenase-1 [1]. IL-10 is mainly expressed by Tregs that have been defined as a specialized subpopulation of T cells that act to suppress activation of the immune system and thereby maintain immune system homeostasis and tolerance to self-antigens [34, 35]. We could in addition in some donors detect IL-10 release in response to the IDO-derived CD4 epitope peptide. Hence, the role of IDO-specific CD4^+^ T cells in immune-regulatory networks may be a complex balance between activation and inhibition depending on the microenvironment. Interestingly, in some donors we detected background IL-10 release in in vitro pre-stimulated ELISPOT assays. This enabled us to recognize that stimulation with the IDO-derived peptide in two healthy donors triggered an overall suppression of IL-10. In this regard, we have previously observed a decrease in IL-10 when IDO-specific CD8^+^ T cells were present [41].

## Clinical perspectives

### Cancer

IDO may exhibit its immune inhibitory functions both in the activation phases (in the draining lymph node) as well as in the effector phases (at the site of the tumor). With regard to the latter, IDO may even by induced as an inflammation-induced counter-regulatory mechanism. Counter-regulatory responses are important in the immune system as they help to limit the intensity and extent of immune responses, which otherwise could cause damage to the host. However, with regard to anti-cancer immunotherapy, counter-regulatory responses antagonize the ability to create an intense immune response against the tumor. Counter-regulation differs from tolerance in the sense that counter-regulation is a secondary event, elicited only in response to immune activation. IDO is known to be induced by both type I and II interferons, which are likely to be found at sites of immune activation and inflammation [31, 36]. In this respect, it should be mentioned that the susceptibility of tumor cells to lysis by IDO-reactive T cells were increased by pre-incubation with IFN-γ [40].

**Fig. 3:**
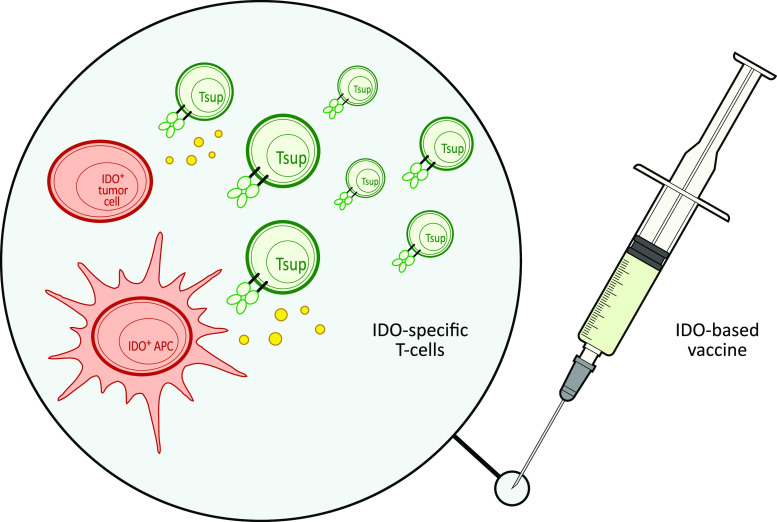
Vaccine induced IDO-specific T cells might kill IDO^+^ suppressive antigen presenting cells (APC) as well as IDO^+^ cancer cells both at the tumor site and in the draining lymph nodes. IDO may exhibit its immune inhibitory functions both in the activation phases (in the draining lymph node) as wells as in the effector phases (at the site of the tumor). Hence, an IDO-based cancer vaccine might work directly at the tumor site by the attack of cancer cells as well as stromal cells as well as in the draining lymph node by the attack of IDO-expressing regulatory cells

Hence, in cancer immune therapy, the boosting of IDO-specific immunity could have both direct and indirect effects (Fig. 3). First of all, IDO-specific, cytotoxic T cells are able to directly recognize and kill IDO^+^ cancer cells. In fact, it may even be speculated that the measurable reactivity to this antigen in normal individuals contributes to immune surveillance against cancer. Furthermore, the induction of IDO-specific immune responses by therapeutic measures could function highly synergistic with additional anti-cancer immune therapy not only by eliminating cancer cells but in addition immune suppressive cells. By definition, anti-cancer immune therapies aim at the induction of an immunological activation and inflammation. The therapy aims to induce as much immune activation as possible (within the limits of acceptable toxicity), and, accordingly, immune suppressive counter-regulation is not desired.

Adoptive transfer of ex vivo expanded tumor-infiltrating lymphocytes (TIL) after host lymphodepletion has the potential to significantly improve the prognosis of patients with metastatic melanoma. The impressive clinical responses associated with adoptive transfer of TIL [9] urge that this strategy is pursued and investigated for the treatment of other types of cancer. In this regard, patient IDO-specific T cells isolated and expanded from PBMC may well be an interesting supplement to the ongoing adaptive T-cell transfer strategies.

It goes without saying that the possible introduction of autoimmunity and toxicity are the major worries when targeting a molecule like IDO. However, the circulation of a measurable number of IDO-specific T cells did not seem to cause autoimmunity. Furthermore, since IDO-specific T cells can be introduced by IFN or CpG this appears to be under solid control. In this regard, an interesting aspect of IDO is that systemic inactivation at the organism level, either pharmacologically or genetically, does not appear to cause autoimmunity [19].

We believe that the findings that presented here justified and warranted clinical testing to evaluate the efficiency and safety of IDO-based vaccinations. Hence, we initiated a phase I vaccination study, which is ongoing (from June 2010) at Center for Caner Immune Therapy, Copenhagen University Hospital, Herlev, in which patients with non-small cell lung cancer (NSCLC) are vaccinated with a IDO-derived peptide with Montanide adjuvant (www.clinicaltrials.gov; NCT01219348).

### Additional pathogenic settings

It has been suggested that IDO may rather be involved in tolerance to non-self-antigens than self-antigens in situations where immune non-responsiveness may be important, for example, during pregnancy [19]. In this respect, induction of IDO^+^ immune-regulatory dendritic cells (DC) have been described to occur during infection of DCs with viruses and intracellular pathogens. In *Listeria monocytogenes* infections, such IDO^+^ DC seems to be involved in protection of the host from granuloma breakdown and pathogen dissemination in advanced human listeriosis. Likewise, it was recently described that IDO is increased in lymph nodes in cutaneous *Leishmania major* infection [17]. IDO is implicated in suppressing T-cell immunity to parasite antigens and IDO inhibition reduced local inflammation and parasite burdens, which suggest that IDO were of benefit for the pathogen, not the host. During HIV infection, multiple mechanisms involving both viral and cellular components contribute to enhance IDO expression and activity in an uncontrolled manner. Among others, HIV inhibits T-cell proliferation by inducing IDO in plasmacytoid DC and macrophages [5]. Furthermore, it was recently described that IDO is increased in hemodialysis (HD) patients compared to healthy donors [10]. Furthermore, IDO suppresses adaptive immunity in HD patients as it is assessed by the response to HBV vaccination. Hence, the targeting of IDO could have synergistic effects in anti-viral immune therapy, for example, in Hepatitis B vaccines.

The fact that IDO may be involved in tolerance to non-self-antigens might have major implications for IDO-based immune therapy as boosting immunity to neoantigens, but not normal self-antigens, by triggering IDO-specific T cells is very attractive. Since IDO-expressing cells might antagonize the desired effects of other immunotherapeutic approaches targeting IDO-expressing cells by vaccination would consequently be easily implementable and highly synergistic with such therapeutic measures. However, it was recently described that although IDO might play biologically important roles in the host response to diverse intracellular infections like *Toxoplasma gondii, leishmaniasis*, and *herpes simplex* virus, the nature of this role that being antimicrobial or immunoregulatory might depend on the pathogen. Hence, IDO inhibition might not always benefit the host. In this regard, IDO inhibition during murine toxoplasmosis led to increased mortality with increased parasite burdens [8]. This should naturally been taken into account when exploring the possible use of IDO-specific T cells in the clinic.

Finally, it should be mentioned that CD14^+^ monocytes are major CMV target cells in vivo. CMV is the most immune dominant antigen to be encountered by the human immune system [43]. Monocytes are responsible for dissemination of the virus throughout the body during acute and late phase of infection. CMV has been shown to induce IDO expression in monocytes, which has been suggested to confer an advantage to CMV-infected monocytes to escape T-cell responses [12]. The CD8^+^ T-cell response to CMV typically comprises a sizeable percentage of the CD8^+^ T-cell repertoire in CMV-seropositive individuals [15]. In light of this, it is possible that IDO-specific T cells might function as support for the constitutive anti-CMV CD8^+^ T-cell response. Naturally, this can only be speculation, but notably we found that the presence of IDO-specific CD4^+^ T-cell responses correlated to the presence of CMV-responses [23].
